# Exploring equity in cancer treatment, survivorship, and service utilisation for culturally and linguistically diverse migrant populations living in Queensland, Australia: a retrospective cohort study

**DOI:** 10.1186/s12939-023-01957-9

**Published:** 2023-09-01

**Authors:** Brighid Scanlon, Jo Durham, David Wyld, Natasha Roberts, Ghasem Sam Toloo

**Affiliations:** 1 Royal Brisbane and Women’s Hospital, Butterfield Street, Herston, QLD 4029 Australia; 2 Queensland University of Technology, 149 Victoria Park Road, Kelvin Grove, Brisbane, QLD 4059 Australia; 3 University of Queensland, St Lucia, QLD 4072 Australia; 4 Surgical, Treatment and Rehabilitation Service, STARS Education and Research Alliance, Herston, QLD 4006 Australia

**Keywords:** Cancer, Health equity, Racialisation, Migrant health, Disparities, Medical oncology

## Abstract

**Background:**

There is strong international evidence documenting inequities in cancer care for migrant populations. In Australia, there is limited information regarding cancer equity for Culturally and Linguistically Diverse (CALD) migrant populations, defined in this study as migrants born in a country or region where English is not the primary language. This study sought to quantify and compare cancer treatment, survivorship, and service utilisation measures between CALD migrant and Australian born cancer populations.

**Methods:**

A retrospective cohort study was conducted utilising electronic medical records at a major, tertiary hospital. Inpatient and outpatient encounters were assessed for all individuals diagnosed with a solid tumour malignancy in the year 2016 and followed for a total of five years. Individuals were screened for inclusion in the CALD migrant or Australian born cohort. Bivariate analysis and multivariate logistic regression were used to compare treatment, survivorship, and service utilisation measures. Sociodemographic measures included age, sex, post code, employment, region of birth and marital status.

**Results:**

A total of 523 individuals were included, with 117 (22%) in the CALD migrant cohort and 406 (78%) in the Australian-born cohort. CALD migrants displayed a statistically significant difference in time from diagnosis to commencement of first treatment for radiation (P = 0.03) and surgery (P = 0.02) and had 16.6 times higher odds of declining recommended chemotherapy than those born in Australia (P = 0.00). Survivorship indicators favoured CALD migrants in mean time from diagnosis to death, however their odds of experiencing disease progression during the study period were 1.6 times higher than those born in Australia (P = 0.04). Service utilisation measures displayed that CALD migrants exhibited higher numbers of unplanned admissions (P = < 0.00), longer cumulative length of those admissions (P = < 0.00) and higher failure to attend scheduled appointments (P = < 0.00).

**Conclusion:**

This novel study has produced valuable findings in the areas of treatment, survivorship, and service utilisation for a neglected population in cancer research. The differences identified suggest potential issues of institutional inaccessibility. Future research is needed to examine the clinical impacts of these health differences in the field of cancer care, including the social and institutional determinants of influence.

## Background

Global migration has increased significantly over the past five decades, with an estimated 281 million international migrants [1]. This is leading to global social, economic, and demographic transformations which have implications for the delivery of equitable healthcare [1]. Disruptive events, such as the COVID-19 pandemic have exposed and amplified the significant health inequities experienced by racialised migrant populations, which are produced and reinforced by widespread social, institutional, and structural determinants [2]. Although health disparities among migrant populations are well documented, previous research has largely homogenised the experiences of migrant groups, without considering their differing backgrounds, experiences or the effects of racialisation [3]. Racialisation is the process whereby racial categories are constructed, leading to different and unequal social, economic and health outcomes [4]. This combination of migrant groups from vastly different social, cultural and linguistic backgrounds leads to research that potentially underestimates the health inequities experienced by racialised migrants. This is exemplified by the misleading ‘Healthy Migrant Effect’ [3, 4], where migrant groups with disparate health determinants, such as voluntary and involuntary migrants, are combined to display misleading, aggregate outcomes [5]. This ‘tyranny of averages’ effectively masks the power differentials, vulnerabilities and health disparities experienced by some migrant groups, such as those affected by racialisation [5]. In response to this, this study focused on ‘Culturally and Linguistically Diverse’ (CALD) migrant populations, who are defined as migrants born in a country or region where English is not the primary language [6]. Whilst the term ‘CALD’ is contested, the authors use it within this paper for consistency in Australian research and to enable comparisons and interpretability [6]. This term was also chosen to acknowledge the unique experiences of racialised migrants in Australia [6, 7].

There is a growing body of work examining the pervasive, and often socially produced health inequities experienced by CALD migrant populations, particularly in complex, chronic diseases, such as cancer [8, 9]. International evidence, whilst often homogenising migrant groups, has identified inequities across the cancer care continuum [10–13]. This includes access to screening services, follow-up, length of survival and quality of treatment [9, 10, 12, 13]. In Australia, inequities among migrant populations are mirrored in some communicable and non-communicable disease, such as cardiovascular disease, mental health disorders and viral hepatitis [14, 15]. In the field of cancer, available evidence suggests that CALD migrants experience inequities in cancer detection, with barriers to access and use of cancer screening programs being well documented, particuarly among women of Middle Eastern and Asian backgrounds [13, 15–17]. Less is known however, about clinical treatment outcomes post-diagnosis, but there is evidence highlighting inequitable treatment quality and poorer quality of life for populations from Middle Eastern, Greek and Chinese backgrounds [18–20]. Inequities have also been observed in the survivorship stage, with reports of continued cancer-related stressors, exacerbated by unmet physical and informational needs for CALD populations [21–23]. Overall, Australian literature remains sparse regarding treatment outcomes of CALD migrants, or their experiences with cancer survivorship and service accessibility. Such limited understanding is a critical barrier to the promotion and operationalisation of health equity within cancer care services in Australia [24]. Additionally, much of the current Australian literature are descriptive, qualitative studies of patient experience, with limited clinical measurement of inequities. To redress this, our study aims quantify and compare equity indicators for cancer treatment, survivorship, and service utilisation between CALD migrant and Australian born cancer cohorts.

## Methods

### Study design and participants

**Fig. 1:**
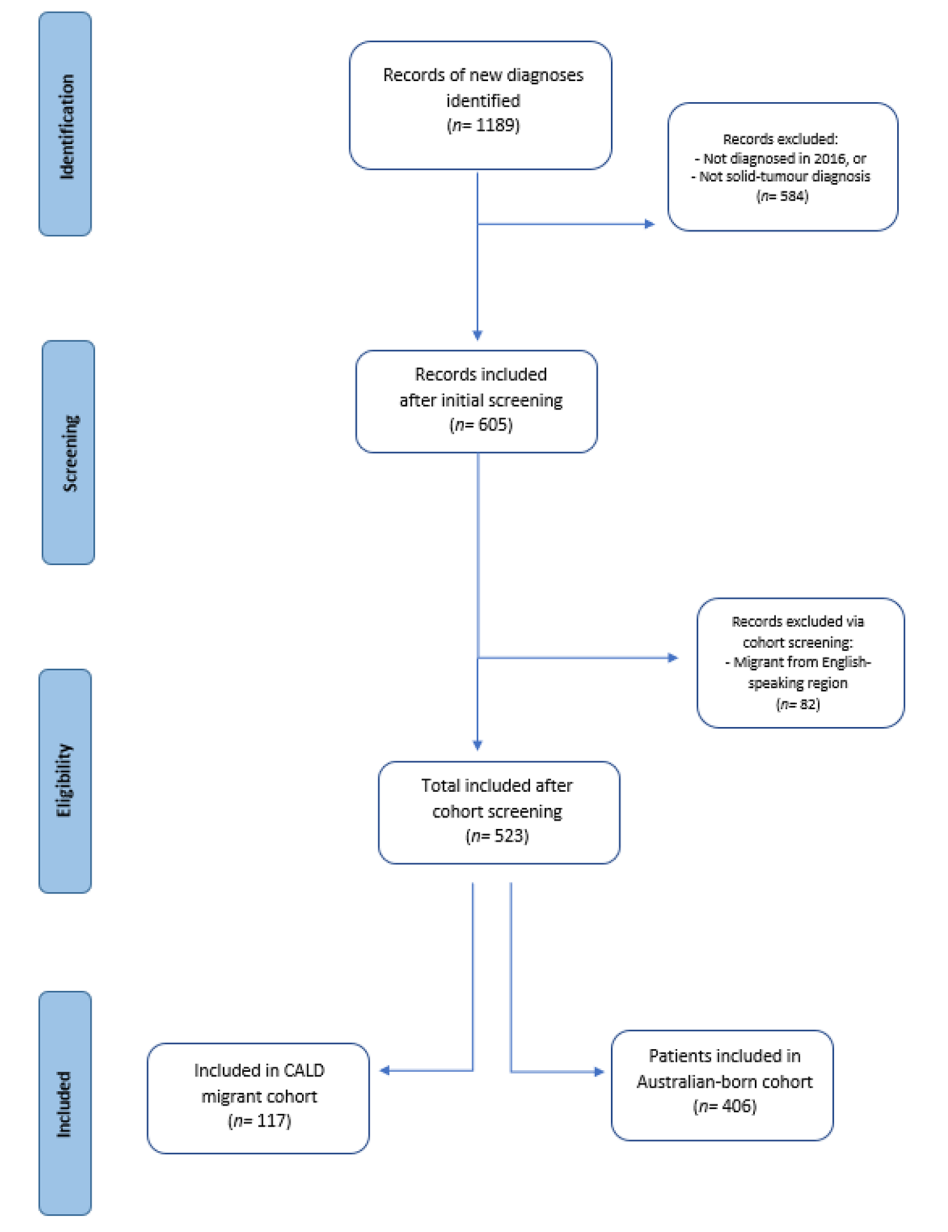
Screening process

This retrospective cohort study screened for inclusion all individuals diagnosed with a solid-tumour malignancy in the year 2016 at a large, tertiary hospital in Queensland, Australia. Using the electronic medical records, participants’ inpatient and outpatient encounters were followed for a total of five years’ post-diagnosis, or until patient death. This study aimed to investigate differences found between CALD migrant and Australian born populations along the cancer care continuum [25]. The inclusion criteria required: 1) diagnosed with a solid tumour malignancy, 2) ≥ 16 years of age at diagnosis. Participants were then disaggregated based on country of birth. Those born in Australia were included in the “Australian born” cohort, regardless of their parents’ backgrounds, and those born outside of Australia were assessed for the “CALD migrant” cohort. Individuals born in a country or region outside of Australia, where English was not the primary language were included in the “CALD migrant” cohort. Māori populations- the Indigenous peoples of Aotearoa (New Zealand)- who spoke a language other than English, such as te reo Māori, were considered CALD and included in the Polynesia region, due to their documented cultural and linguistic diversity [26]. Individuals born in New Zealand who did not identify as Māori were excluded from the study. Those who had migrated from countries or regions where English was the primary language and the participants’ primary language was also English, such as England, were excluded from the study. The screening process is displayed in Fig. 1. Ethical approval for this study was sought and obtained by the facility prior to commencing data collection: (HREC/2021/QRBW/74,613).

### Data sources and variables

A data collection tool based on the National Cancer Control Indicators (NCCIs), developed by Cancer Australia, was created [27]. The NCCIs are a list of clinical measures that signify significant treatment outcomes or disease status [27]. There are a number of measures for each stage of the cancer care continuum. These indicators were used in the absence of standardised equity indicators and with the knowledge that they have been used previously to highlight cancer disparities in vulnerable populations, such as Aboriginal and Torres Strait Islander Australians [27]. This study utilises measures for the diagnosis, treatment and survivorship phases, with findings from the prevention and detection phases reported separately [28]. In addition to the NCCIs, a range of sociodemographic and service utilisation variables were collected from the routinely collected data in the electronic medical records. This study was limited by data availability within the electronic medical records.

#### Independent variables

The key independent variable was CALD migrant status. Participants who were diagnosed with a solid-tumour malignancy in the year 2016 were screened for inclusion in the CALD migrant or Australian born cohort. These variables were created in order to compare the NCCIs and service utilisation variables between the two cohorts. These variables were dichotomised into a binary variable exhibiting CALD migrant status or not. Other independent variables collected included the sociodemographic variables of participants age (continuous), sex (male, female), region of birth (Australian born or respective region of birth), marital status (married, de facto, separated, never married or widowed), religion, post code (metropolitan, regional, or rural) and employment status (employed, unemployed or retired). Post code data were coded per the Australian government’s Rural, Remote and Metropolitan Area (RRMA) classification [29]. All sociodemographic variables were collected based on availability of routinely collected data in the electronic medical records, per patient registration forms.

### Dependent variables

Based on availability of data, as of January 2016, data were extracted for all participants for a total of five years’ follow up, or until patient death. Dependent variables included the NCCIs, which were a mixture of categorical and continuous measures. Those associated with treatment included radiation, chemotherapy, and surgery use (yes, no), time from diagnosis to first treatment (radiation, chemotherapy, or surgery) commencement (continuous), clinical trial use (yes, no), whether participants declined recommended chemotherapy (yes, no). The NCCIs associated with survivorship included recurrence within the study period (yes, no), disease progression within the study period (yes, no), five-year survival (yes, no) and time from diagnosis to death (continuous). Health utilisation measures were also extracted based on data availability. These were cumulative, continuous measures spanning the study period. These included number of unplanned admissions (continuous), length of unplanned admissions (continuous), number of emergency department visits (continuous) and failure to attend appointments (continuous). Interpreter usage was also examined as interpreter required (yes, no) and documented interpreter usage (yes, no).

### Statistical analysis

Data were analysed using IBM Statistical Package for the Social Sciences Version 26 [30]. Sociodemographic, NCCIs and service utilisation measures were observed for both cohorts and compared. Bivariate associations between categorical variables were analysed with Chi Square Test or if indicated, the Fisher’s Exact Test. ANOVA (with F-test) was used to identify associations between categorical and continuous variables. Variables found to be associated in the bivariate analyses (P = < 0.1) were then explored in multivariate analyses using logistic regression (for categorical dependent variables) and ANCOVA (for continuous dependent variables). The significance value was set at < 0.05. Post hoc analysis, such as Tukey’s HSD was not utilised as there were fewer than three groups to compare.

## Results

### Sociodemographic characteristics

A total of 1189 participants were screened over a three-month period (1st August 2021-1st November 2021). Of those, 523 met inclusion criteria for the study, with 117 (22%) included in the CALD migrant cohort and 406 (78%) in the Australian born cohort. The mean age of participants was 59 (SD = 13.4) and almost 56% were female, with a higher proportion of females in the CALD migrant cohort (66% vs. 53%). The most common regional backgrounds for those in the CALD migrant cohort were Asia (32%), Europe (32%) and Polynesia (27%). Most participants lived in a metropolitan area, with less than 10% of CALD migrants, and less than 30% of the Australian born cohort residing in rural or regional areas. Twenty-one (*n* = 21) participants indicated that they required an interpreter, of which, 43% (*n* = 9) had no record of interpreter usage throughout the five-year study period. Participants’ sociodemographic characteristics are presented in Table 1.

**Table 1:** Sociodemographic characteristics

Characteristic	*CALD Cohort* *n = 117*			*Australian-born cohort* *n = 406*		
	*n*	*%*	*Mean ± SD*	*n*	*%*	*Mean ± SD*
**Age**	117		57.4 ± 15.5	406		59.5 ± 12.7
**Sex**						
Male	40	34.2		192	47.3	
Female	77	65.8		214	52.7	
**Region of birth**						
Australian-born	0	0		404	99.5	
First Nation’s Australian	0	0		2	0.5	
Polynesia	31	26.5		0	0	
South America	3	2.6		0	0	
Europe	37	31.6		0	0	
Asia	37	31.6		0	0	
Africa	5	4.3		0	0	
Middle East	4	3.4		0	0	
**Marital Status**						
Married	68	58.1		183	45.1	
De facto	9	7.8		39	9.6	
Separated	19	16.2		79	19.5	
Never married	11	9.4		68	16.7	
Widowed	10	8.5		35	8.6	
**Religion**						
Christian	47	40.2		165	40.6	
No religion	59	50.4		238	58.6	
Ratana	2	1.7		0	0	
Buddhist	2	1.7		1	0.2	
Bahai	1	0.9		0	0	
Hindu	3	2.6		1	0.2	
Echkankar	0	0		0	0	
Mormon	1	0.9		0	0	
Judaism	1	0.9		1	0.2	
Muslim	1	0.9		0	0	
**Post code**						
Metropolitan	106	90.6		286	70.4	
Regional	7	6.0		80	19.7	
Rural	4	3.4		40	9.9	
**Employment status**						
Employed	54	46.1		156	38.4	
Unemployed	14	12.0		46	11.3	
Retired	49	41.9		198	48.8	

### Treatment indicators

Table 2 displays only statistically significant bivariate relationships of CALD status with cancer treatment, survivorship, and service utilisation measures. Mean time from diagnosis to commencement of first treatment (radiation, chemotherapy, or surgery) were explored through a one-way ANOVA (Table 2). This revealed that there was a statistically significant difference between mean time from diagnosis to first treatment (radiation) commencement between the two cohorts (F = 3.63; P = 0.030). There was also a statistically significant difference between time from diagnosis to first treatment (surgery) between the cohorts (F = 4.90; P = 0.028). Bivariate analysis revealed a statistically significant relationship between CALD migrant status and declining recommended chemotherapy (P < 0.00). Of those, (n = 17) who declined recommended chemotherapy, 82.4% (n = 14) were from the CALD migrant cohort. This was further explored in logistic regression models (Table 3), which displayed that the odds of a CALD migrant declining recommended chemotherapy were 16.6 times higher than those born in Australia (CI 4.61–59.67), when controlling for sex. There were no statistically significant differences found between the two cohorts for frequency of chemotherapy, radiation or surgery use, clinical trial use or time from diagnosis to first treatment (chemotherapy) commencement (results not displayed).

**Table 2:** Bivariate analysis

Variable	Measure	CALD *n* (*%)*	AUS *n* (*%)*	CALD Mean ± SD	AUS Mean ± SD	Test	P Value
Treatment							
*Diagnosis to first treatment (radiation)*	Time (months)			1.96 (2.60)	1.42 (0.73)	F = 3.63	**0.030**
*Diagnosis to first treatment (surgery)*	Time (months)			0.46 (0.66)	0.25 (0.62)	F = 4.90	**0.028**
*Declined recommended chemotherapy*	Yes No	14 (14.1) 85 (85.9)	3 (0.9) 315 (99.1)			X^2^ = 33.63	**< 0.001**
Total *n*		99	318				
**Survivorship**							
*Diagnosis to death*	Time (months)			22.73 (15.19)	17.22 (12.43)	F = 6.89	**0.009**
*Progression during study period*	Yes No	66 (60.0) 44 (40.0)	205 (51.4) 194 (48.6)			X^2^ = 8.835	**0.011**
Total *n*		110	399				
**Service utilisation**							
*Unplanned admissions*	Cumulative total (admissions)			4.03 (3.45)	3.19 (2.56)	F = 8.08	**0.005**
*Length of unplanned admissions*	Cumulative total (days)			26.99 (38.42)	18.06 (18.53)	F = 12.08	**< 0.001**
*Failure to attend appointments*	Cumulative total (appointments missed)			2.47 (7.46)	0.81 (2.42)	F = 14.72	**< 0.001**

Notes: Only variables with statistically significant associations with sociodemographic factors (P = < 0.10) were included in the multivariate analyses

* Logistic Regression ** ANCOVA

**Table 3:** Results of multivariate analysis (Logistic regression and ANCOVA)

Variable (Reference)*	Measure	OR	95% CI		P value
**Declining recommended chemotherapy (No)**					
CALD status (CALD migrant)	Yes/No	16.573	4.604–59.665		**0.000**
Sex (Male)		0.769	0.253–2.337		0.643
**Disease progression (No)**					
CALD status (CALD migrant)	Yes/No	1.584	1.017–2.469		**0.042**
Employment (Employed)		1.668	1.374–2.026		**0.000**
Variable (Covariate)**	Measure	**CALD ** Mean ± SD	**AUS ** Mean ± SD	**F**	P value
**Diagnosis to death** **(Age)**	Time (months)	22.69	17.23	F = 6.80	**0.010**

Notes: Only variables with statistically significant associations with sociodemographic factors (P = < 0.10) were included in the multivariate analyses

* Logistic Regression ** ANCOVA

### Survivorship indicators

One-way ANOVA revealed a statistically significant difference between mean time from diagnosis to death, with CALD migrants surviving longer (F = 6.89; P = 0.00), displayed in Table 2. An analysis of covariance was undertaken (ANCOVA), which showed this difference remained when controlling for age (F = 6.80; P = 0.01), Table 3. Disease progression was assessed through multivariate logistic regression, revealing the odds of a CALD migrant experiencing progression during the study period were 1.6 times higher than Australian born participants (CI 1.02–2.47), when controlling for employment (P = 0.04), Table 3. There were no statistically significant differences found between cohorts for five-year survival or frequency of disease recurrence (results not displayed).

### Health service utilisation

One-way ANOVA was undertaken for the service utilisation measures. This displayed a statistically significant difference between the means of these cohorts, Table 2. CALD migrant populations had a higher mean number of unplanned admissions (F = 8.08; P = < 0.00). The mean length of these unplanned admissions was also statistically significantly higher for CALD migrants (F = 12.08; P = < 0.00). Failure to attend appointments was also statistically significantly higher in the CALD migrant cohort (F = 14.72; P = < 0.00). Multivariate logistic regression was not undertaken as there were no statistically significant bivariate associations with the sociodemographic variables identified.

## Discussion

Current literature tells us that health inequities are systematic, unfair and avoidable differences in the opportunities groups have to attain optimal health and health outcomes [31]. It is recognised that healthcare institutions play an integral role in the production of health inequities, through the unequal distribution of power, resources, and providing privileged positioning to the majoritarian healthcare model [32, 33]. There is strong evidence that institutions can foster marginalisation and structural disadvantage for racialised populations, evidenced as differences in outcomes and access to services [4, 34]. This study demonstrates the need for equity research that spans the cancer continuum and considers the effects of racialisation [15, 32]. Novel findings presented include differences in time from diagnosis to first treatment (radiation and surgery) commencement, higher odds of declining recommended chemotherapy, higher odds of disease progression, higher unplanned admissions, and longer length of those admissions for CALD migrants. The study also identified a significant survival difference, favouring CALD migrant populations. Findings from this study underscore the critical importance of centring the role of institutions in the production of health inequities, as many of the key differences between cohorts related to access and use of health services.

The treatment phase displayed statistically significant differences in time from diagnosis to first treatment (radiation and surgery) commencement for CALD migrants, and over 16 times higher odds of CALD migrants declining a recommended chemotherapy. These findings are highly concerning due to the established relationships between delayed or declined anticancer therapies, and disease progression [35–37]. Delays to treatment commencement have been reported internationally, with studies in Australia and North America finding that delays perceived as avoidable occurred more frequently in the secondary care setting [38–40]. The literature has highlighted the importance of establishing national benchmarks for time-to-treatment that are reflective of contemporary treatments [38]. Additionally, there is a need to streamline primary, secondary and tertiary care services [40, 41] .

International evidence reports ‘racial differences’ in chemotherapy uptake, particularly in African American communities [42, 43], however Australian data are sparse and inconsistent. An Australian retrospective single-centre analysis of 211 patients with early-stage breast and colorectal cancer, revealed no statistically significant difference between CALD and non-CALD populations in the uptake of adjuvant chemotherapy [44]. Conversely, a larger Australian retrospective study of 19, 453 participants revealed that chemotherapy trial participation was significantly lower among CALD populations (5.7% vs 8.4%; P = 0.001) [45]. In response to these findings, Australian research has identified the critical need to expand use of interpreter services and for the development of translated trial and treatment-related information sheets to aid in equitable treatment uptake [45].

A potential contributor to differences in treatment use were the differences in tumour stream, with CALD migrants making up a higher proportion of breast (30% vs. 19%) and gynae-oncology (14% vs. 9%) tumours, and Australian born participants making up a higher proportion of head and neck tumours (21% vs. 8%). This suggests a critical need to investigate potential treatment delays and barriers to chemotherapy uptake among individual tumour streams for CALD populations in Australia.

In the survivorship phase, the mean length of survival was longer for CALD migrants, with CALD migrants surviving an average of five months longer during the study period. The reason for this is unclear, however both Australian and international evidence has displayed differences in the incidence and mortality of certain cancer types amongst migrant groups [15, 46] and has highlighted the need to disaggregate data by both tumour type and migrant group. Future research should utilise subgroup analysis to assess if this longer length of survival for CALD migrant populations can be reproduced in individual tumour streams.

CALD migrants were more likely to experience progression of their disease during the study period. In Australia, the 5-year relative survival for all cancers is 70% from diagnosis [47] and consequently, a longer period of follow up may be required to adequately assess equity in overall survival. In the absence of mature data on overall survival, progression-free survival has been used as a surrogate outcome measure [48, 49]. Progression-free survival has been shown to have a positive correlation with overall survival, however the statistical significance of this varies considerably between tumour type and lines of treatment [48]. To investigate the clinical significance of shorter progression-free survival, larger studies that allow for subgroup analysis are required [50]. Future research should also investigate the significance of the relationship between declining recommended chemotherapy and shorter progression-free survival for CALD migrant populations.

In this study, service utilisation measures were used as a proxy measure for healthcare accessibility. Findings displayed that CALD migrants had more unplanned admissions and a longer cumulative length of those admissions. The mean cumulative length of admissions was significantly longer for CALD migrants at 23 vs. 17 days for those born in Australia. These results concur with international literature which identifies racial background as a predictor of unplanned hospital admissions and increased length of admissions [51, 52]. Importantly, both unplanned admissions and length of stay have been linked to increased mortality and hospital expenditure [51, 52]. These findings may be influenced by the higher proportion of Australian born participants living in rural and remote areas (30% vs. 10%), underestimating their service utilisation. This is an important consideration due to the established impacts of rural and remote geography on healthcare access [53]. It is imperative that health systems and researchers explore the causes and impact of unplanned hospital admissions and length of stay for CALD migrants, whilst considering the impact of geographic location on service use.

This study suggests there may be issues with institutional accessibility at both entry and exit points of the hospital setting [54]. This argument is strengthened by the finding that CALD migrants were more likely to fail to attend scheduled outpatient appointments. This has implications for continuity of care, access to timely and appropriate care and patient outcomes [55]. This also highlights the need for stronger relationships between health services and CALD communities. These relationships are necessary to build trust, reduce disparities and promote equitable healthcare [55].

One potential barrier to accessible health services identified in this study was the low use of interpreters. Almost half (43%) of those who identified requiring an interpreter on admission did not have any record of interpreter use throughout the five-year study period. This included during diagnostic discussions, chemotherapy and other treatment education, and discussions regarding prognosis and preferences at end of life. It is unclear if patients did not receive an interpreter, if they used a family member as an interpreter, or if interpreter usage was simply not documented. This is important because if patients are not receiving a timely and appropriate interpreter, this significantly threatens informed consent and equitable access to information for CALD patients. There is a need for consistent and clear documentation of interpreter usage, including usage of family members, if we are to promote equitable access to healthcare information and fulfil patients’ right to interpreter services [56].

The findings demonstrate the need for standardised equity indicators, so that cancer outcomes can be easily measured and compared in a reproducible way. This study demonstrates that Cancer Australia’s NCCIs, although not comprehensive, can provide insights into inequities across the cancer continuum. More comprehensive health equity indicators, which consider the complexity of the cancer journey and reflect the values and preferences of CALD communities are needed for health institutions to adequately assess their progress towards equitable health service provision.

Given the critical role health institutions play in promoting health equity, it is imperative that future research considers the way in which racialised health inequities are institutionally produced and can thus be influenced by structural change. Health institutions have the opportunity to play a significant role in the reduction of health disparities and promotion of health equity, through adapting the healthcare model to reflect the needs of all individuals and communities they serve.

### Limitations

The sample size of this study was insufficient to conduct subgroup analysis of tumour streams or migrant groups. This limitation means that although this study focused on CALD migrant populations, it did not account for the diversity within the included CALD populations. Additionally, this study did not provide comparison between CALD and non-CALD migrant populations. This would be a valuable comparison in future research, to assess the relative contribution of both ethnicity and migrant status. Similarly, subgroup analysis of tumour streams would allow for more robust clinical interpretations of health differences. This lack of subgroup analysis limits generalisability and application of findings to the wider Queensland population. The health utilisation measures may have been influenced by the higher proportion of the Australian born cohort living in rural or remote locations (30% vs. 10%). This may have reduced the follow-up of participants who accessed services in their local areas during the study period. An important limitation was the inability to assess the impact of participants’ socio-economic status, due to limitations in the electronic medical records. It must also be acknowledged that health services are only able to assess ‘realised’ health access and are unable to assess ‘potential’ health access [54]. This means that only those who are engaged with health services are represented within this study.

## Conclusions

In conclusion, this study has provided novel insights into treatment, survivorship, and service utilisation outcomes for CALD migrant populations. These findings have identified several areas in which CALD migrant populations may experience cancer-related inequities. Future research is required to examine the clinical implications of these differences in the field of cancer care and the institutional determinants that influence these differences.

## Data Availability

Data are available from the corresponding author on reasonable request: brighid.scanlon@hdr.qut.edu.au.

## List of abbreviations

CALD: Culturally and Linguistically Diverse
NCCIs: National Cancer Control Indicators
